# Intestinal Microbiota Protects against MCD Diet-Induced Steatohepatitis

**DOI:** 10.3390/ijms20020308

**Published:** 2019-01-14

**Authors:** Kai Markus Schneider, Antje Mohs, Konrad Kilic, Lena Susanna Candels, Carsten Elfers, Eveline Bennek, Lukas Ben Schneider, Felix Heymann, Nikolaus Gassler, John Penders, Christian Trautwein

**Affiliations:** 1 Department of Internal Medicine III, University Hospital RWTH Aachen, 52074 Aachen, Germany; Kai.Markus.Schneider@gmail.com (K.M.S.); amohs@ukaachen.de (A.M.); konrad.kilic@gmail.com (K.K.); lcandels@ukaachen.de (L.S.C.); celfers@ukaachen.de (C.E.); ebennek@ukaachen.de (E.B.); Lukas.Ben.Schneider@gmx.de (L.B.S.); fheymann@ukaachen.de (F.H.); 2 Department of Pathology, Klinikum Braunschweig, 38118 Braunschweig, Germany; n.gassler@klinikum-braunschweig.de; 3 Department of Medical Microbiology, School of Nutrition and Translational Research in Metabolism, Maastricht University Medical Center, 6200 MD Maastricht, The Netherlands; j.penders@maastrichtuniversity.nl

**Keywords:** NASH, Gut-liver-Axis, microbiota, MCD

## Abstract

Non-alcoholic fatty liver disease (NAFLD) is the most common liver disease in western countries, with a continuously rising incidence. Gut-liver communication and microbiota composition have been identified as critical drivers of the NAFLD progression. Hence, it has been shown that microbiota depletion can ameliorate high-fat diet or western-diet induced experimental Non-alcoholic steatohepatitis (NASH). However, its functional implications in the methionine-choline dietary model, remain incompletely understood. Here, we investigated the physiological relevance of gut microbiota in methionine-choline deficient (MCD) diet induced NASH. Experimental liver disease was induced by 8 weeks of MCD feeding in wild-type (WT) mice, either with or without commensal microbiota depletion, by continuous broad-spectrum antibiotic (AB) treatment. MCD diet induced steatohepatitis was accompanied by a reduced gut microbiota diversity, indicating intestinal dysbiosis. MCD treatment prompted macroscopic shortening of the intestine, as well as intestinal villi in histology. However, gut microbiota composition of MCD-treated mice, neither resembled human NASH, nor did it augment the intestinal barrier integrity or intestinal inflammation. In the MCD model, AB treatment resulted in increased steatohepatitis activity, compared to microbiota proficient control mice. This phenotype was driven by pronounced neutrophil infiltration, while AB treatment only slightly increased monocyte-derived macrophages (MoMF) abundance. Our data demonstrated the differential role of gut microbiota, during steatohepatitis development. In the context of MCD induced steatohepatitis, commensal microbiota was found to be hepatoprotective.

## 1. Introduction

Non-alcoholic fatty liver disease (NAFLD) is the most common liver disease in western societies and due to the obesity epidemic the incidence keeps rising [1,2,3]. The term NAFLD covers a spectrum of disease manifestations ranging from liver steatosis over non-alcoholic steatohepatitis (NASH), liver fibrosis, to advanced disease states, such as cirrhosis and hepatocellular carcinoma (HCC) [2]. Western sedentary lifestyle and high caloric diets are the strongest and most significant risk factors for NAFLD development [4]. Accordingly, 90% of obese patients are affected by hepatic steatosis, which usually remains clinically asymptomatic. Thirty percent of patients diagnosed with NAFLD demonstrate histological signs of inflammation, which causes liver cell damage and fuels disease progression towards liver fibrosis and more advanced states, such as cirrhosis and HCC [5]. Given the pivotal role of hepatic inflammation as a mediator of disease phase transition towards irreversible cirrhosis and HCC, understanding the underlying mechanisms that perpetuate the inflammatory response in the liver seems key, in order to design novel disease-modifying therapies.

Recent data identify infiltrating innate immune cells, such as monocyte-derived macrophages (MoMFs) and neutrophil granulocytes, as mediators of the hepatic inflammation, during NASH [6,7,8,9]. Pharmacological inhibition of the MoMF infiltration ameliorates NASH development, in man and mice [9,10]. These cells express high levels of intracellular and extracellular pathogen recognition receptors (PRRs) and recognize damage-associated molecular patterns (DAMPs) released upon tissue damage, as well as pathogen- or microbiota-associated molecular patterns (PAMPSs/MAMPs) that reach the liver, via the portal circulation [11,12]. In NLRP3 and NLRP6 inflammasome deficient mice, unfavorable intestinal microbiota has been linked to a loss of intestinal barrier integrity and increased translocation of MAMPs into the liver, where they activate TLR4- and TLR9-mediated hepatic inflammation [13].

These data indicate that translocation of bacterial products from the gut into the liver, contribute to liver inflammation during NASH. In humans, unfavorable gut microbiota composition has been identified, both as a regulator of body weight and body-fat composition, as well as a decisive factor in the intestinal barrier impairment [14]. Obese individuals have significantly increased levels of small intestinal bacterial overgrowth (SIBO), compared to healthy lean subjects, and may suffer from increased gut permeability, prompting a translocation of lipopolysaccharides (LPS) [15,16,17].

Similarly, mice fed with a high-fat diet become obese, develop insulin resistance, and demonstrate intestinal barrier impairment and increased translocation of LPS into the portal vein—a phenotype that closely reflects the disease mechanisms of human NASH [18].

Methionine-choline-deficient (MCD) diet represents another well-established rodent model of non-alcoholic steatohepatitis, which results in hepatic steatosis, oxidative stress, inflammation, and fibrosis [19]. On the one hand, a lack of choline in this diet, hampers the export of triglycerides (TG) via a very low-density lipoprotein (VLDL) packaging from hepatocytes, resulting in hepatic steatosis [20]. On the other hand, the essential amino acid methionine is required for the synthesis of S-adenosylmethionine (SAM) and glutathione, which are both antioxidants [21]. Although the data suggest an important role of sterile inflammation and the innate immune response, mediated by PRR signaling in MCD-induced NASH [13,22,23], it is a matter of debate as to what extent gut microbiota and gut-liver crosstalk contribute to steatohepatitis development in this model.

Here, we investigated the relevance of gut microbiota in MCD-induced experimental NASH.

## 2. Results

### 2.1. Microbiota Depletion Augments Steatohepatitis Development in the Murine MCD Model

**Figure 1:**
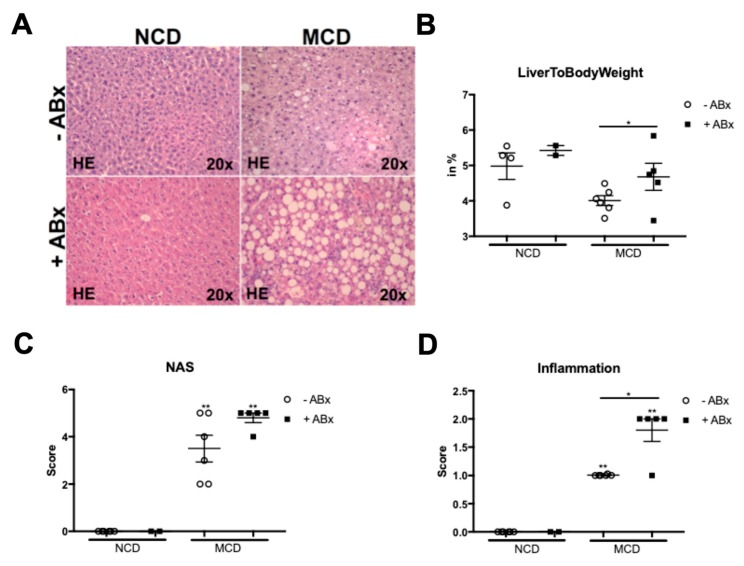
Antibiotic treatment (ABx) aggravates non-alcoholic steatohepatitis (NASH) in the murine methionine-choline deficient (MCD) model. (**A**) Representative liver histology (hematotoxylin and eosin staining) showing livers of the wild-type (WT) mice with (+ABx) and without (–ABx) treatment on the normal chow diet (NCD) and after MCD treatment. (**B**) Increased “Inflammation” score in the ABx treated mice. (**C**) *ABx* treated mice had a higher histopathological non-alcoholic fatty liver disease (NAFLD) activity score (NAS). (**D**) ABx resulted in significantly increased Liver-to-Body-Weight ratios. Data are expressed as the mean ± SD from 2–5 mice per group and were considered significant if * *p* < 0.05, ** *p* < 0.01.

To investigate the relevance of gut microbiota for the development and progression of MCD induced steatohepatitis, 8 weeks old male mice were fed a methionine choline-deficient (MCD) diet for 8 weeks. One group of mice received a well-established cocktail of four non-absorbable broad-spectrum antibiotics in their drinking water, for the whole feeding experiment, while the other group of age and gender-matched mice received normal drinking water. After 8 weeks of dietary intervention, the caecum of antibiotics-treated mice was strongly enlarged. As expected, liver hematoxylin and eosin-stained liver sections of the MCD-fed mice displayed all hallmarks of NASH, including steatosis, inflammation, and fibrosis (Figure 1A). Interestingly and other than expected, antibiotic treatment (ABx) resulted in aggravated steatosis and significantly increased the inflammatory cell infiltration (Figure 1A,B). In addition, the *ABx*-treated mice had a higher histopathological NAFLD activity score (NAS) and a significantly higher liver-to-body weight ratio (Figure 1C,D).

Altogether, these data indicated that microbiota depletion results in a more severe liver injury.

### 2.2. Antibiotic Treatment Increases Hepatic Fat Accumulation in the MCD-Fed Mice, But Is Not Associated with a Metabolic Phenotype Characteristic of the Human NASH

**Figure 2:**
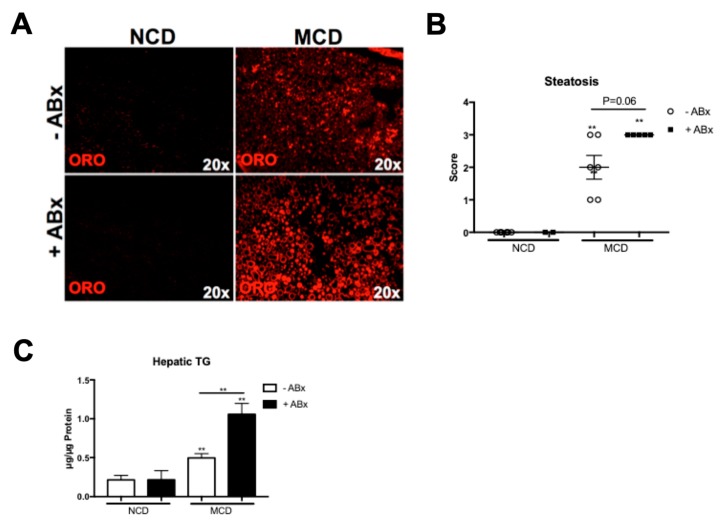
Antibiotic treatment increased the hepatic fat accumulation in the MCD-fed mice. (**A**) Representative Oil Red O stainings demonstrated increased the hepatic lipid accumulation upon an antibiotic treatment. (**B**) Steatosis score was higher in the ABx-treated mice, compared to the MCD-fed control mice. (**C**) Colorimetric hepatic triglyceride assay confirmed significantly increased hepatic triglycerides (TG) levels in +ABx group. Data are expressed as the mean ± SD from 2–5 mice per group and were considered significant if ** *p* < 0.01.

As previously reported, the MCD treatment resulted in a significant loss in the total body weight and MCD feeding did not trigger increased fasting glucose levels. Interestingly, the ABx-treated mice demonstrated an increased hepatic lipid accumulation shown by HE and Oil Red O stainings (Figure 1A and Figure 2A), which was also reflected in the histopathological steatosis score (Figure 2B). A colorimetric hepatic triglyceride assay confirmed the significantly increased triglyceride levels in the ABx-treated mice, compared to the MCD-treated control mice (Figure 2C). Hence, antibiotic treatment in the MCD-fed mice prompted an increased hepatic lipid storage, but was not associated with the metabolic phenotype characteristic of the human NASH.

### 2.3. Microbiota Depletion Augments the Inflammatory Response During the MCD-Induced Steatohepatitis

**Figure 3:**
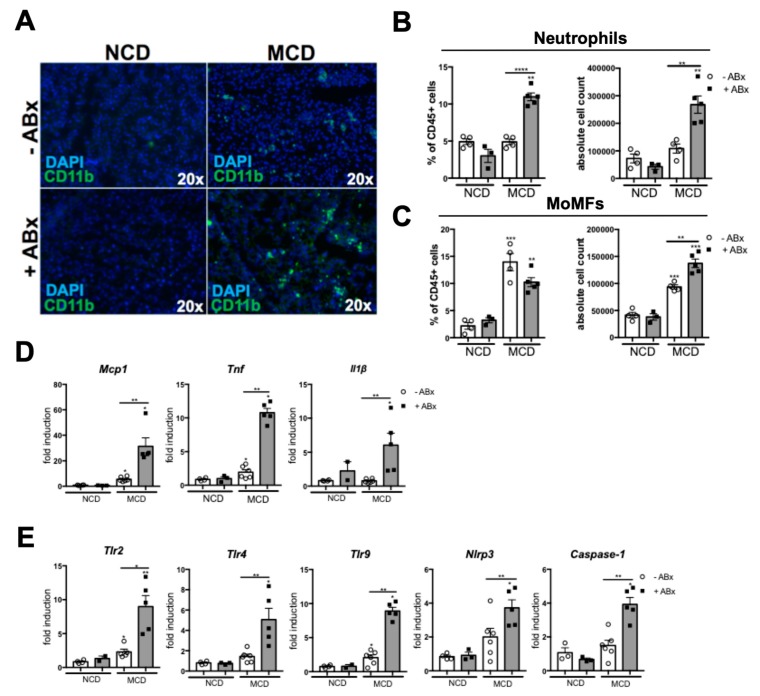
Microbiota depletion augments the inflammatory response during the MCD-induced steatohepatitis. (**A**) Representative immunofluorescence staining against CD11b, showing an increased infiltration of the CD11b+ cells in the Abx group. (**B**) Flow cytometry (FACS) shows increased infiltration of the neutrophils (CD11b+ Ly6G+ living leukocytes) after antibiotic treatment. (**C**) Monocyte-derived macrophages (MoMFs) (defined as CD11b^hi^ F4/80+ living leukocytes) abundance is lower in the ABx group, compared to the MCD-fed control mice. (**D**) Pro-inflammatory mRNA expression of the Mcp, Tnf, and Il1beta. GAPDH was used as a housekeeping gene. (**E**) ABx treatment prompted pronounced mRNA expression of pathogen recognition receptors (PRRs), including Tlr2, Tlr4, Tlr9, Nlrp3, and Caspase-1. GAPDH was used as a housekeeping gene. Data are expressed as the mean ± SD from 2–5 mice per group and were considered significant if * *p* < 0.05, ** *p* < 0.01, *** *p* < 0.001. **** *p* < 0.0001.

Next, we investigated the relevance of intestinal microbiota for hepatic inflammation, during the MCD-induced steatohepatitis. The Abx-treated mice demonstrated significantly higher inflammation in the histopathological analyses of the HE sections (Figure 1B). To further analyze which cell type mediated the pronounced inflammatory response, we first performed immunofluorescence staining against the CD11b. Here, CD11b+ immune cells were increased in the livers of the Abx-treated mice, compared to the MCD-fed controls (Figure 3A). To further dissect which cell type accounted for the inflammatory response, we performed a flow cytometry (FACS) analysis of the liver homogenates. MCD feeding induced a strong infiltration of the MoMFs (defined as Ly6G-, CD11b^hi^, F4/80^low^). In contrast, both, the absolute and relative numbers of neutrophil granulocytes (defined as CD11b+, Ly6G+) were only slightly increased in the MCD versus the NCD-fed mice (Figure 3B,C). While antibiotic treatment only slightly augmented the MoMF infiltration in the MCD-fed mice (Figure 3C), neutrophil granulocytes showed a significant almost two-fold increase in the MCD + ABx group, compared to the MCD−Abx group, which was reflected, both, in the absolute, as well as relative cell numbers (Figure 3B). Together, these data demonstrated that antibiotic treatment in the MCD-induced steatohepatitis triggered neutrophil infiltration.

The inflammatory response was orchestrated by a significantly increased mRNA expression of pro-inflammatory genes, such as monocyte chemotactic protein 1 (*Mcp1*), tumor necrosis factor alpha (*Tnf*), as well as interleukin 1 beta (*Ilβ*) in the Abx-treated mice, compared to the control mice (Figure 3D). Interestingly, this phenotype was also associated with a pronounced expression of PRRs, including toll like receptor 2 (*Tlr2),* toll like receptor 4 *(Tlr4),* toll like receptor 9 *(Tlr9),* NLR family, pyrin domain containing 3 (*Nlrp3)* and *Caspase-1*, which have all been implicated in the NASH pathogenesis (Figure 3E).

Together, these data demonstrated that microbiota depletion in the MCD-fed mice unleashes a strong hepatic inflammatory innate immune response, which is mediated by the neutrophil granulocytes.

### 2.4. Intestinal Microbiota Protects against Excessive Liver Fibrosis

**Figure 4:**
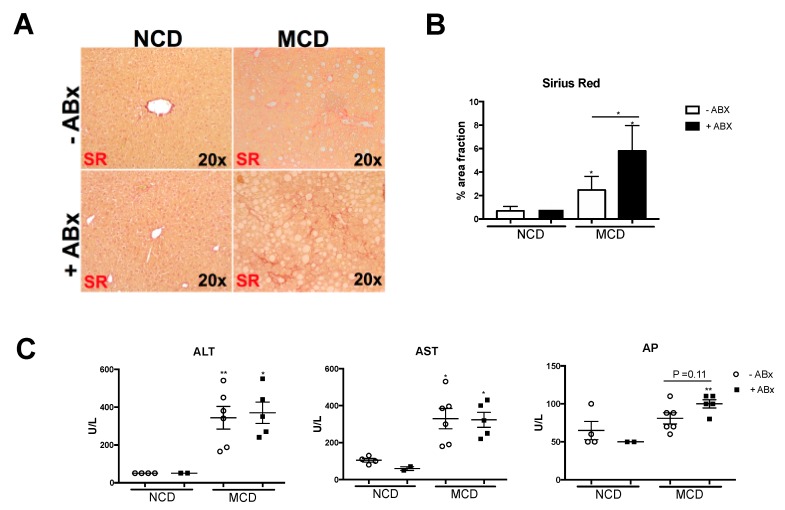
Antibiotic treatment fuels excessive liver fibrosis in experimentally-induced MCD-NASH. (**A**) Representative Sirius red staining of the liver sections showing the collagen fibers in red. (**B**) Quantification of the Sirius Red positive area, using the ImageJ software (at least 5 areas in 100× magnification per mouse). (**C**) Serum liver function tests. Data are expressed as the mean ± SD from 2–5 mice per group and were considered significant if * *p* < 0.05, ** *p* < 0.01.

Hepatic inflammation might lead to the activation of hepatic stellate cells, which transdifferentiate into myofibroblasts-facilitating collagen deposition and, thus, contribute to tissue remodeling and disease progression towards liver fibrosis. Next, we sought to investigate whether the increased liver inflammation upon antibiotic treatment also translated into aggravated liver fibrogenesis. Indeed, the depletion of the intestinal microbiota was associated with a strong increase in liver fibrosis, as evidenced by the Sirius red stainings (Figure 4A). Histopathological quantification of collagen fibers revealed an about 2,5-fold increase in the Sirius Red positive area (Figure 4B). Serum liver transaminases Alanin-aminotransferase (ALT) and aspartate-aminotransferase (AST) were both significantly increased after 8 weeks of the MCD treatment. While AST and ALT did not increase upon antibiotic treatment, alkaline-phosphatase (AP) levels were higher in the +Abx group, compared to the MCD-fed control animals (Figure 4C). In sum, these data showed that antibiotic treatment prompted excessive liver fibrosis in the experimental MCD-induced NASH.

### 2.5. MCD Diet Impacts the Intestinal Homeostasis and Microbiota Composition

**Figure 5:**
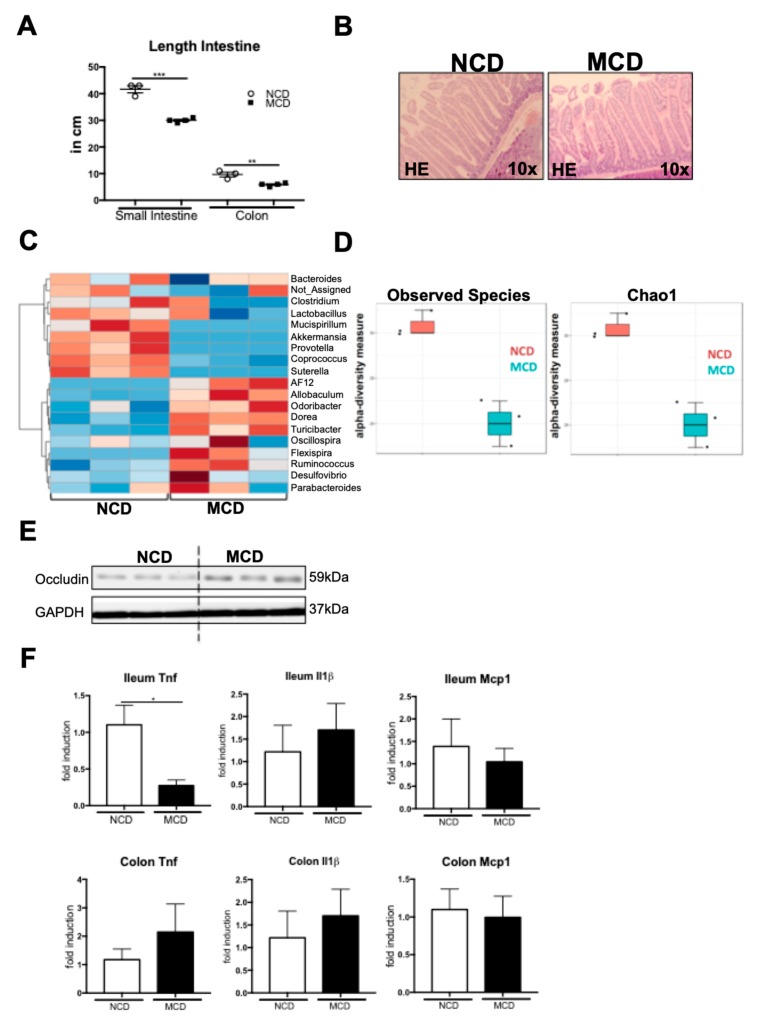
MCD diet impacts intestinal homeostasis and microbiota composition. (**A**) MCD diets leads to shortening of small and large intestines. (**B**) Representative histology of the paraffin-fixed duodenum sections. (**C**) Clustered heatmap analysis of the microbiota composition of the normal chow or the MCD-fed mice. (**D**) “Observed species” and “Chao1” alpha diversity metrics were reduced, upon MCD feeding. (**E**) Occludin protein levels in the ileum tissue lysates. (**F**) Tnf, Il1beta, and Mcp1 mRNA expression, determined by the qRT-PCR in the ileum and the colon samples. * *p* < 0.05, ** *p* < 0.01, *** *p* < 0.001.

Steatohepatitis has been linked to intestinal dysbiosis. After eight weeks of MCD feeding, the small intestines, as well as the colons, were atrophic and significantly shorter than those of the NCD-fed control mice (Figure 5A). This phenotype was also reflected in the HE histology, which demonstrated a shortening of the intestinal villi in the duodenum of the MCD-fed mice (Figure 5B). To investigate the microbiota composition, we collected cecal microbiota samples to isolate the metagenomic DNA and performed a 16s ribosomal gene (rDNA) amplicon sequencing of the V1–V3 hypervariable region, using the 454 platform. Eight weeks of MCD treatment resulted in marked alterations in the microbiota composition (Figure 5C). Among the genera that were differentially regulated between the NCD- and MCD-fed mice, we identified a decrease in the potentially probiotic *Lactobacillus*, as well as *Akkermansia*, and an increase in the *Ruminococus*, which has been linked to liver fibrosis (Figure 5C) [24]. Along with changes in the individual bacterial communities, MCD feeding resulted in a strong overall decrease of the microbiota alpha diversity metrics, such as observed species, as well as Chao1 (Figure 5D). Although MCD feeding induced changes in the microbiota composition and a loss of species richness, we did not observe a major decrease in the Occludin tight junction expression in the ileum of the MCD-fed mice (Figure 5E). While the mRNA expression of *Tnf* in the ileum was even significantly decreased upon MCD feeding, other inflammatory genes, including the *Il1b* and *Mcp1* were unaffected, both in the ileum and the colon (Figure 5F).

Together, these data demonstrate that the MCD diet impacts the intestinal microbiota composition and prompts both macroscopic and microscopic changes in intestinal architecture. This phenotype was not associated with a strong suppression of tight junctions or increased inflammatory gene expression.

## 3. Discussion

Non-alcoholic Steatohepatitis (NASH) is a disease characterized by hepatic steatosis and inflammation, which can further progress to fibrosis and HCC [1,25]. NASH is strongly associated with obesity and the metabolic syndrome, and due to the obesity epidemic in western societies, the incidence of NASH is rising [26]. Over- and malnutrition is widely accepted as the main cause of NASH—however, not all obese Patients develop NASH and at the same time there are lean patients suffering from active NASH [27]. This observation indicates that there must be additional mechanisms, e.g., genetic or other environmental factors, which drive the transition from simple steatosis to NASH [28,29,30].

Recent data demonstrated that gut-liver communication and gut microbiota represent important modulators of the liver disease [12,30,31,32]. Intestinal microbiota composition of NASH patients is significantly different from healthy individuals and an emerging body of preclinical data support a causal role of the gut microbiota in NASH development [24,33]. Patients suffering from NASH may develop an intestinal barrier impairment, facilitating increased translocation of PAMPs and MAMPs, through the portal vein into the liver [16]. Maintenance of the intestinal homeostasis and barrier integrity relies on a complex interaction of the host immune system and commensal microbiota, which may be hampered by environmental factors and affected by host genetics [34]. There is a huge body of experimental evidence showing that depletion of the intestinal microbiota by antibiotic treatment or in Germ-free (GF) mice, protects from a high-fat diet or western-style-diet induced NASH [18,35,36,37]. These dietary regimens nicely reflect human NAFLD, caused by the western sedentary lifestyle—the mice become overweight, and develop insulin resistance and fatty liver disease [38,39]. In contrast to the high-fat diet (HFD) feeding, mice fed with an MCD diet, actually loose body weight, and do not develop insulin resistance [39]. Mechanistically, choline deficiency impairs the VLDL synthesis and hepatic lipid export. Body and liver weight loss in the MCD model is induced by an increased energy expenditure, without increased food consumption [40]. In our study, antibiotic treatment resulted in pronounced liver remodelling and collagen deposition in the MCD-fed mice, which was also reflected in an increased liver-to-bodyweight ratio, in these mice. The role of the intestinal microbiota in MCD-induced steatohepatitis, is still a matter of debate. We, and other researchers have shown that microbiota depletion using broad-spectrum antibiotics, protects mice from HFD or western-style, diet-induced NASH [18]. Contrasting these data obtained in the HFD or the western-style diet (WSD) models, we showed here that microbiota depletion in the MCD-fed mice, augments steatohepatitis, by unleashing a strong, innate-immune response, orchestrated by neutrophil infiltration. In our study, MCD feeding prompted intestinal dysbiosis, encompassing a reduced microbiota alpha diversity, likely reducing probiotic bacteria, and inducing intestinal macroscopic, as well as microscopic, structural changes. Still, a complete depletion of microbiota did not reverse the intestinal shortening and even exacerbated steatohepatitis.

While our data clearly showed that a complete depletion of microbiota is detrimental in the MCD model, Hanao-Mejia et al. demonstrated that a dysbiotic microbiota of the inflammasome-deficient mice conferred susceptibility to the MCD-induced NASH, which was communicable via co-housing. In contrast, the probiotic microbiota modulation has beneficial effects both in the HFD model, as well as in the MCD-induced NASH. The VSL#3 probiotic treatment attenuated liver fibrosis in the MCD-fed mice, without affecting the steatohepatitis and hepatic steatosis, by upregulation of the anti-fibrotic transforming growth factor β (TGF-β) pseudoreceptor, Bambi [41].

Collectively, these data suggest a pathogenic role of the gut microbiota not only in the HFD models, but also in the MCD-induced steatohepatitis. While our data showed that a complete depletion of microbiota, exacerbates liver disease, an unfavorable microbiota composition might also drive the disease progression and probiotic microbiota modulation might have a therapeutic potential.

There is a good body of evidence demonstrating that antibiotic treatment with Glycopeptid, Aminopenicillin, Aminoglycosid, and Nitroimidazole, results in an almost complete depletion of the intestinal microbiota [42,43,44]. As previously shown, mice receiving antibiotics showed a massively enlarged caecum, which has also been described in the GF mice. Yet, we cannot exclude that the antibiotic treatment caused an overgrowth with certain antibiotic resistant bacteria or fungi, which might account for the observed phenotype.

Based on our current data, we can only speculate why an antibiotic treatment has such opposing effects in the MCD model, compared to the HFD or the WSD treatment. Similar to our findings, a beneficial role of the commensal microbiota, in preventing murine CCL4-induced liver fibrosis has been shown [45]. Increased liver fibrosis was observed in the germ free (GF) mice, compared to the conventional mice. Various pathogen recognition receptors (PRRs) signal via the Myd88/Trif and a genetic deficiency of this important signaling node prompted a similar phenotype to what was observed in the GF animals. Toll like receptor 2 (TLR2) and Toll like receptor 5 (TLR5) signal upstream of the Myd88. While it has been shown that genetic deletion of the TLR2 and the TLR5 is associated with enhanced steatohepatitis, upon MCD feeding [46,47], in the choline-deficient, I-amino-acid defined (CDAA) dietary model and HFD models, TLR2 and TLR9 deletion were protective [48,49,50]. Interestingly, the TLR4 deficiency conferred a partial protection against NASH, in both the HFD and MCD models [23,51].

Gut microbiota is strongly shaped by diet and represents an important source of the TLR ligands. Compositional changes of microbiota and TLR ligands might explain the differential impact of the various TLR pathways, depending on the dietary model [52].

In future studies, it might be interesting to investigate the role of the gut microbiota at different time points—during early inflammatory initiation versus a later progression towards fibrosis. Additionally, data on the MCD-induced steatohepatitis development in germ free animals or using different regimens of antibiotic treatment, would complement our study.

A better understanding of how microbiota-mediated signals shape the hepatic inflammatory response during steatohepatitis development and progression, might guide future, targeted, microbiota modulation therapies.

## 4. Materials and Methods

### 4.1. Mice Experiments

All animal experiments were approved by the appropriate German authorities (LANUV, North Rhine-Westphalia, Az 84-02.04.2012.A260, approved 03/26/2013). All animals received humane care, according to the criteria outlined in the “Guide for the Care and Use of Laboratory Animals”, prepared by the National Academy of Sciences, and published by the National Institutes of Health (NIH publication 86-23 revised 1985). C57BL/6 wild-type (WT) mice (C57BL/6 background) were housed in filter-top cages. Mice that were 6–8 week old, were included in the experiments.

For 8 weeks, the mice were fed with the methionine-choline deficient diet (MCD (960439), MP Biomedicals, Heidelberg, Germany). A normal chow diet (NCD) (provided by the Animal Facility at the University Hospital RWTH Aachen, Germany) was used as a control diet.

Tissue and blood collection, RNA isolation, triglyceride measurement in liver tissue, cDNA synthesis, real-time quantitative polymerase chain reaction, and measurement of serum parameters (aminotransferases, glutamate dehydrogenase and alkaline phosphatase) were performed as described previously [53,54].

### 4.2. Administration of the Broad-Spectrum Antibiotics

Eradication of intestinal microbiota in rodents, was performed using a well-established cocktail of four broad-spectrum antibiotics (0.5 g/L Neomycin, 1 g/L Metronidazol, 1 g/L Vancomycin, 1 g/L Ampicillin). Antibiotic treatment was initiated 2 weeks prior to the start of the experimental diet, in 6 weeks old male mice. Antibiotics were administered in the drinking water for the whole dietary feeding period and were changed every second day.

### 4.3. Immunofluorescence Analysis

Fixation of slides was performed in 4% PFA at room temperature. 5 µm liver cryosections were stained with rat anti-mouse CD11b (BD Biosciences, Heidelberg, Germany).

Fluorescence signal was obtained using a secondary antibody conjugated with Cy3 (Jackson Immunoresearch, West Grove, PA, USA). Mounting solution containing DAPI (Vector Laboratories, Burlingame, CA, USA) was used to counterstain the nuclei of hepatocytes.

### 4.4. Microbiota—16S rRNA V1-V3 Amplicon Library Preparation

In order to extract the metagenomic DNA, 200mg of the cecal content were mechanically homogenized. After a collum-based purification, the PSP SPIN Stool DNA plus kit (Stratec Molecular GmbH, Berlin, Germany) was used to isolate the microbial DNA. The frozen cecal content was added to sterile vials filled with Lysis buffer (Stratec Molecular, Berlin, Germany), 0.5 g of 0.1 mm zirconia/silica beads (BioSpec, Bartlesville, OK, USA), and four 3.0–3.5 mm glass beads (BioSpec). Alternately, by keeping the samples for one minute, on ice, in between the cycles, the samples were homogenized in a Magna Lyser device (Roche, Basel, Switzerland), for one minute, at a speed of 5500 rpm three times. The samples were isolated, afterwards, using the PSP SPIN Stool DNA plus kit and, according to the manufacturer’s instructions, eluted in a final volume of the 200 µL. A barcoded sense primer, consisting of the 454 Titanium platform A linker sequence (5′-CCATCCCTGCGTGTCTCCGACTCAG-3′), a key (barcode) which was unique for each sample, the 16S rRNA 534R primer sequence 5′-ATTACCGCGGCTGCTGG-3′, and a reverse primer consisting of a 9:1 mixture of two oligonucleotides, 5′-B-AGAGTTTGATCMTGGCTCAG-3′, and 5′-B-AGGGTTCGATTCTGGCTCAG-3′, where B represents the B linker (5′-CCTATCCCCTGTGTGCCTTGGCAGTCTCAG-3′), followed by the 16S rRNA 8F and 8F-Bif primers, was used to generate the amplicon libraries for pyrosequencing of the 16s rDNA V1-3 regions.

For the PCR amplification, we used 1x FastStart High Fidelity Reaction Buffer, 1.8 mM MgCl2, 1nM dNTP solution, 5U FastStart High Fidelity Blend Polymerase (from the High Fidelity PCR System (Roche, Indiapolis, IN, USA)), 0.2 mM reverse primer, 0.2 mM of the barcoded forward primer (unique for each sample), and 1 µL of template DNA. The following thermal cycling conditions were used—an initial denaturation (94 °C, 3 min), followed by 25 cycles of denaturation (94 °C, 30 s), annealing (51 °C, 45 s), extension (72 °C, 5 min), and a final elongation step (72 °C, 10 min). Using the AMPure XP purification (Beckman Coulter, Brea, CA, USA), subsequently, the amplicons were purified, as instructed by the manufacturer, before elution in 1x low TE (10 mM Tris-HCl, 0.1 mM EDTA, pH 8.0). To determine the concentration we applied the Quant-iT PicoGreen dsDNA reagent kit (Invitrogen, New York, NY, USA), using a Victor3 Multilabel Counter (Perkin Elmer, Waltham, MA, USA). To ensure proportional representation of each sample, the amplicons were mixed in equimolar concentrations. The 454 sequencing run was performed on a GS Junior pyrosequencing system, using Titanium chemistry (Roche, Branford, CT, USA).

### 4.5. Microbiota—Data Analysis

To minimize the error rate, raw pyrosequencing reads were passed through quality filters, using Mothur version 1.32. For the further analysis, we retained only sequences matching the following criteria—perfect proximal primer fidelity, a minimum average score of 25, over a window size of 50 nucleotides, a read length between the 200 and 590 nucleotides, a maximum of one ambiguous base call, and a maximum homopolymer length of 6. The data were further analyzed using Quantitative Insights Into the Microbial Ecology (QIIME) version 1.8 [55]. After de-multiplexing, sequences were clustered by the UCLUST [56] algorithm into operational taxonomic units (OTUs), based on a 97% sequence similarity against the Greengenes reference set version May 2013 [57]. The default parameters for the UCLUST were applied, with the exception of the following parameters—maxrejects = 100 and stepwords = 16. The influence of the pyrosequencing errors was minimized by disabling the creation of the de novo OTUs for sequences that did not cluster to the reference sequences.

Observed OTUs (observed richness) and Chao1 index (estimated richness) have been calculated as the metrics of species richness and diversity, within the communities (alpha-diversity). *MicrobiomeAnalyst* was used for the hierarchical clustering and heatmap visualization, Ward’s Clustering was performed on Genus level using the Euclidean distance [58].

### 4.6. Histology

For the tissue sections, 4% paraformaldehyde (PFA) was used as a fixative; these section were then embedded in paraffin. For the hematoxylin and eosin (HE) staining, we cut the liver tissue into 2 µm thick sections. The staining then got reviewed by a board certified pathologist, whose scoring was performed following a modified algorithm established for NASH, referred as the NAS-score [59]. Hepatocellular lipid deposits were scored in relation to the liver cells, with droplets (score 0: <5%; 1: 5–33%; 2: 33–66%; 3: >66%), and histologically, the inflammatory tissue activity was evaluated in a three-level score (no inflammatory focus: 0; 1: 1; 2–4: 2; >4: 3) while a two-level score (0; 1) categorized the degree of hepatocellular ballooning. Additionally, the paraffin-embedded tissue sections were stained with Sirius Red, in order to evaluate the fibrosis development, as described previously [18].

### 4.7. Flow Cytometry Analysis of the Intrahepatic and Intestinal Leukocytes

Leukocytes were isolated from the fresh liver tissue, as previously described [18]. Liver leukocytes were stained with 7-AAD, CD45, CD11b, CD11c, F4/80, Ly6G, and Ly6C. All samples were acquired by flow cytometry (FACS Fortessa; BD Biosciences) and analyzed using the Flowjo software (Tree Star Inc., Ashland, OR, USA).

### 4.8. Statistical Analysis

Data are expressed as the mean ± standard error of the mean. Statistical significance was determined via one-way analysis of variance, followed by a student’s *t*-test.
